# Improving Capacity Assessments for Treatment Refusal in Psychiatry

**DOI:** 10.1192/bjo.2026.11380

**Published:** 2026-06-30

**Authors:** Samukeliso Fundira, Mohamed Jalloh, Vasudevan Krishnan

**Affiliations:** St George’s Hospital, Stafford, United Kingdom

## Abstract

**Aims:**

Adult patients are expected to understand, retain, weigh and use information to make a decision about their illness. When patients refuse recommended medical investigations, clinicians have a clinical, ethical, and legal responsibility to assess and record decision-making capacity. Failure to do so may compromise patient safety and expose clinicians and organisations to medico-legal risk. This quality improvement project aimed to assess whether decision-making capacity assessments were documented when psychiatric patients declined standard admission investigations, which included physical examinations, electrocardiograms (ECGs), and blood tests.

**Methods:**

A cross-sectional audit was conducted involving 18 adult patients admitted over the audit period. Data was collected retrospectively from clinical records and reviewed to determine patient acceptance or refusal of admission investigations and whether decision-making capacity assessment was documented at the time of refusal. Patients who accepted all investigations were included to provide context for overall practice, with focused analysis on those who refused one or more components of physical examination, ECG, or blood tests.

**Results:**

13 of the 18 patients agreed to all admission investigations. Five patients refused at least one component of the recommended investigations. Among these five patients, there was no documented evidence of a decision-making capacity assessment at the time of refusal, resulting in a 0% documentation rate. This result revealed a significant gap between expected standards of care and current clinical practices. The absence of recorded capacity assessments raises concerns regarding informed refusal, patient autonomy, and medico-legal accountability. Possible contributory factors include time pressures in acute settings, limited clinician awareness of documentation requirements, and the absence of structured prompts within clinical documentation systems.

**Conclusion:**

This audit demonstrated inadequate documentation of capacity assessments when patients refused admission investigations. Targeted quality improvement interventions are required such as, improving clinician education on capacity assessments, documentation practices, incorporation of capacity prompts into admission proformas, and re-audit to assessimprovement. Strengthening documentation of capacity assessments will support ethical clinical decision-making, safeguard patient autonomy and reduce medico-legal risk.

